# Short-term leprosy forecasting from an expert opinion survey

**DOI:** 10.1371/journal.pone.0182245

**Published:** 2017-08-16

**Authors:** Michael S. Deiner, Lee Worden, Alex Rittel, Sarah F. Ackley, Fengchen Liu, Laura Blum, James C. Scott, Thomas M. Lietman, Travis C. Porco

**Affiliations:** 1 Francis I. Proctor Foundation, University of California, San Francisco, San Francisco, California, United States of America; 2 Department of Ophthalmology, University of California, San Francisco, San Francisco, California, United States of America; 3 Yale University, School of Public Health, New Haven, Connecticut, United States of America; 4 University of California, Berkeley, California, United States of America; 5 Department of Mathematics and Statistics, Colby College, Waterville, Maine, United States of America; 6 Department of Epidemiology and Biostatistics, University of California, San Francisco, San Francisco, California, United States of America; University of Massachusetts, UNITED STATES

## Abstract

We conducted an expert survey of leprosy (Hansen’s Disease) and neglected tropical disease experts in February 2016. Experts were asked to forecast the next year of reported cases for the world, for the top three countries, and for selected states and territories of India. A total of 103 respondents answered at least one forecasting question. We elicited lower and upper confidence bounds. Comparing these results to regression and exponential smoothing, we found no evidence that any forecasting method outperformed the others. We found evidence that experts who believed it was more likely to achieve global interruption of transmission goals and disability reduction goals had higher error scores for India and Indonesia, but lower for Brazil. Even for a disease whose epidemiology changes on a slow time scale, forecasting exercises such as we conducted are simple and practical. We believe they can be used on a routine basis in public health.

## Introduction

Leprosy (Hansen’s disease) is a chronic infectious disease which has been the target of WHO control programs aimed at elimination of leprosy as a public health burden [1, 2]. Caused by *Mycobacterium leprae* [3], a slowly growing agent closely related to the tubercle bacillus [4], leprosy today is highly curable with WHO combination therapy [5]. In addition, the BCG vaccine, widely used against tuberculosis, appears to elicit partial protection against leprosy, providing additional control [6, 7].

Current leprosy control targets, as envisaged by the WHO, are (a) to have no grade 2 disability among pediatric patients, (b) to reduce the number of new leprosy cases with grade 2 disability to less than one case per million population, and (c) for no countries to have legislation allowing leprosy-related discrimination [8]. Current goals also recommend monitoring of the annual new case detection rate; transmission of leprosy underlies the persistence of the disease—and resulting disability—in populations.

India publishes leprosy statistics at the state/territory level, including the annual new case detection and new case detection rate [9, 10]. Moreover, the WHO has provided recent world totals as well as the number of cases for leading countries, including India, Brazil, and Indonesia [11]. As part of a recent expert survey, our group asked experts to forecast the number of cases of leprosy by state/territory in India, as well as the total number of cases for the world, and for India, Brazil, and Indonesia [12]. Expert opinion is important, not only for the obvious reason that expert opinion drives policy, but because expert opinion could incorporate specific knowledge about the epidemiology and surveillance of leprosy to improve forecasts. Indeed, a human expert-based forecasting platform was recently applied to US influenza forecasting [13]. Recent years have seen increased interest in epidemic forecasting in a number of settings [14–17]. The survey provides us an opportunity to compare statistical short-term forecasts with these expert opinion forecasts [18].

## Materials and methods

## Methods

### Expert opinion

#### Survey methods

We devised a cross-sectional survey for individuals with expertise in leprosy, neglected tropical diseases, or forecasting. Leprosy experts were identified by searching PubMed for articles published in or after 1995 containing terms leprosy, leprae, or Hansen’s disease in the title or abstract. Experts in neglected tropical diseases were identified by collecting email addresses from all articles published in the journal *PLoS Neglected Tropical Diseases* (excluding leprosy experts). Finally, forecasting experts were identified from PubMed searches as discussed in the Appendix. Duplicates were removed; individuals in the leprosy group were not included in the neglected tropical disease group, and neither were included in the forecasting expertise group. Finally, email addresses for individuals associated with the authors’ research groups were removed. The 11-item survey was implemented in Qualtrics, and sent in February 2016. Questions included demographics, an expert assessment question, assessment of the chance that the 2020 goals will be met, and forecasting questions. UCSF Institutional Review Board approval was granted, and per recommendations, any user was allowed to opt out of any question.

The experts were asked to anonymously answer several demographic questions, including whether they posessed a medical degree and for what country each had the greatest expertise. The experts also provided a subjective probability that global “interruption of transmission” would be achieved by 2020, and a probability that the goal of reducing the incidence of new grade 2 disability below 1 per million would be achieved by 2020. Full discussion of these responses is provided elsewhere [12]. We included one question for validation or expert assessment, in which the experts were asked whether tuberculoid or lepromatous leprosy was more likely to correspond to the paucibacillary classification.

Experts were then asked to forecast the next reported case count for the world, and for the top three countries reporting cases: Brazil, India, and Indonesia. To increase the number of forecast targets, we also asked respondents to forecast reported case counts for the states and territories of India. India was chosen because of its consistent public reporting and because of the large population (leading to relatively large case counts despite India’s successes in leprosy control). Each expert was presented with data for six randomly chosen states and territories, to keep the survey of manageable length. From the questionnaire, the respondents provided us with 95% credible intervals and a median forecast. We considered direct elicitation of a full probability distribution for the forecast targets (world, India, Brazil, Indonesia, and states in India) to be impractical due to time limitations in taking an online survey. We only asked each expert for their median forecast, and for a lower 2.5% and upper 97.5% bound. The survey instructions indicated these were to be interpreted in a Bayesian sense: the probability that the true value is less than the lower bound is 2.5%, and so forth. Each respondent was asked for leprosy new case detection forecasts for the world, for India, Brazil, and Indonesia (total), and for a randomly chosen set of 5–6 Indian states.

#### Derivation of probabilistic forecast

For each target, we used the three numbers provided to produce a full probabilistic distribution as follows. Let *L* be the lower bound, *M* be the median, and *U* be the upper bound, and let Δ_−_ = *M* − *L* and Δ_+_ = *U* − *M*. Let *s*_−_ = Δ_−_/*Z*_*α*/2_ and *s*_+_ = Δ_+_/*Z*_*α*/2_, where *Z*_*α*/2_ ≈ 1.96 is the upper 97.5% quantile of the standard normal distribution. We assumed the distribution had support on the interval [*M* − 5*s*_−_, *M* + 5*s*_+_]. We found the unique quadratic spline passing through the points (*M* − 5*s*_−_, 0), (*L*, 0.025), (*M*, 0.5), (*U*, 0.975), and (*M* + 5*s*_+_, 1) that minimizes the total integrated square of the curvature. This was used as the estimated cumulative density function, and computed separately for each expert, for each forecast. We computed the probability of every possible integer observation that could be reported for each state or country. This process yielded a probabilistic forecast for each individual expert. We also computed the forecast mean from this distribution. Finally, these were averaged together, yielding a pooled ensemble probabilistic forecast.

### Statistical forecasts

#### Regression

Simple linear regression was used for the time series for reported leprosy cases for the world, from 2005–2014. The data were log transformed, and then time series bootstrap was conducted [19], with a fixed window of 2.

For forecasting the incidence in India, Brazil, and Indonesia, we proceeded as follows. We used data from 2005–2014 as reported by the WHO, for the top 20 countries (excluding, however, the Democratic Republic of the Congo, due to political unrest). Statistical forecasts were conducted using linear mixed effects regression [20]. The data were log-transformed (with zeros being treated as 0.5 for transformation), and a model with both random slopes and random intercepts was chosen. The Metropolis algorithm was used to explore the parameter space of this model (with five parameters: the overall intercept, an overall slope by time, the random intercept variance, random slope variance, and residual variance). The chain was initialized at the maximum likelihood estimate of the model. Conditional on choices for these parameters, simulation from the conditional distribution of the random effects given the data was used to yield an ensemble of realizations for each point in the MCMC-derived sample. Conversion to probabilistic forecasts was constructed by smoothing the histogram of simulated case numbers (for new case detection forecasts). All 19 countries were used in fitting the model, though forecasts were only reported for India, Brazil, and Indonesia.

Similar methods were used for the India state-level forecasts. These were conducted using the data from 2008 to present, by state or territory, using data published by the Indian National Leprosy Eradication Programme [9, 10, 21–33]. New case counts for 2008–2015 are reported by the Indian NLEP (with year 2008 corresponding to the twelve month period ending March 31, 2008, and so on).

#### Short term trend

In practice, regression methods for forecasting do not explicitly discount past observations in general. Such models may be insensitive to recent trend changes. We computed Holt-Winters forecast paths [34] for each leprosy case series for the period 2006–2014, using the log transformed series. The values of the two Holt-Winters smoothing coefficients *λ*_0_ and *λ*_1_ [34] which minimized the squared error were chosen for the world case counts and for the Brazil, India, and Indonesia time series. For the India state and territory data, we fit Holt-Winters coefficients to each log transformed series (replacing zeros with 0.5). The average *λ*_0_ and *λ*_1_ over all 35 states and territories were used in forecasting 2015. To construct standard errors based on a short series, we implemented time series bootstrap resampling with a fixed window of 2 using residuals from ordinary least squares regression [19]. These were used to generate resampled data sets to which the Holt-Winters procedure could be applied to generate one step ahead forecasts. The mean and standard deviation of these were used to produce the final forecast errors on the transformed scale.

#### Scoring

Probabilistic forecasts were scored using the log-likelihood of future data (ignorance score) [35]. We also report the absolute error (a measure which is not, however, a proper score). Forecast errors were computed for each individual expert as well as for the ensemble estimate. The expert forecasts were developed prior to the publication of the most recent data used in evaluation and were thus masked. Evaluation data for the world, for Brazil, India, and Indonesia were obtained from the WHO [36]. India state data were obtained from the Indian NLEP [37]. Data used for evaluation were never used in fitting.

After computing the individual expert forecasts, we computed the absolute error for each expert (the difference between his or her predicted mean, and the subsequent observed value). We used the Wilcoxon rank sum test to assess the relationship between the absolute forecast error and the following binary variables: whether or not the respondent reported having a medical degree, whether or not the respondent claimed India or claimed Brazil as the country for which they had the greatest expertise, and whether or not the respondent answered correctly the expertise assessment question. We used ordinary least squares regression to assess the relationship between the absolute forecast error and the following continuous predictors: the elicited probability of meeting the 2020 goals for global “interruption of transmission” and for reducing incident grade 2 disability to less than 1 new case per million. Note that standard errors are produced by bootstrap, and *P*-values by Monte Carlo permutation testing.

All statistical analysis was conducted using R v. 3.2.1 for MacIntosh (R foundation for Statistical Computing, Vienna, Austria).

## Results

**Fig 1:**
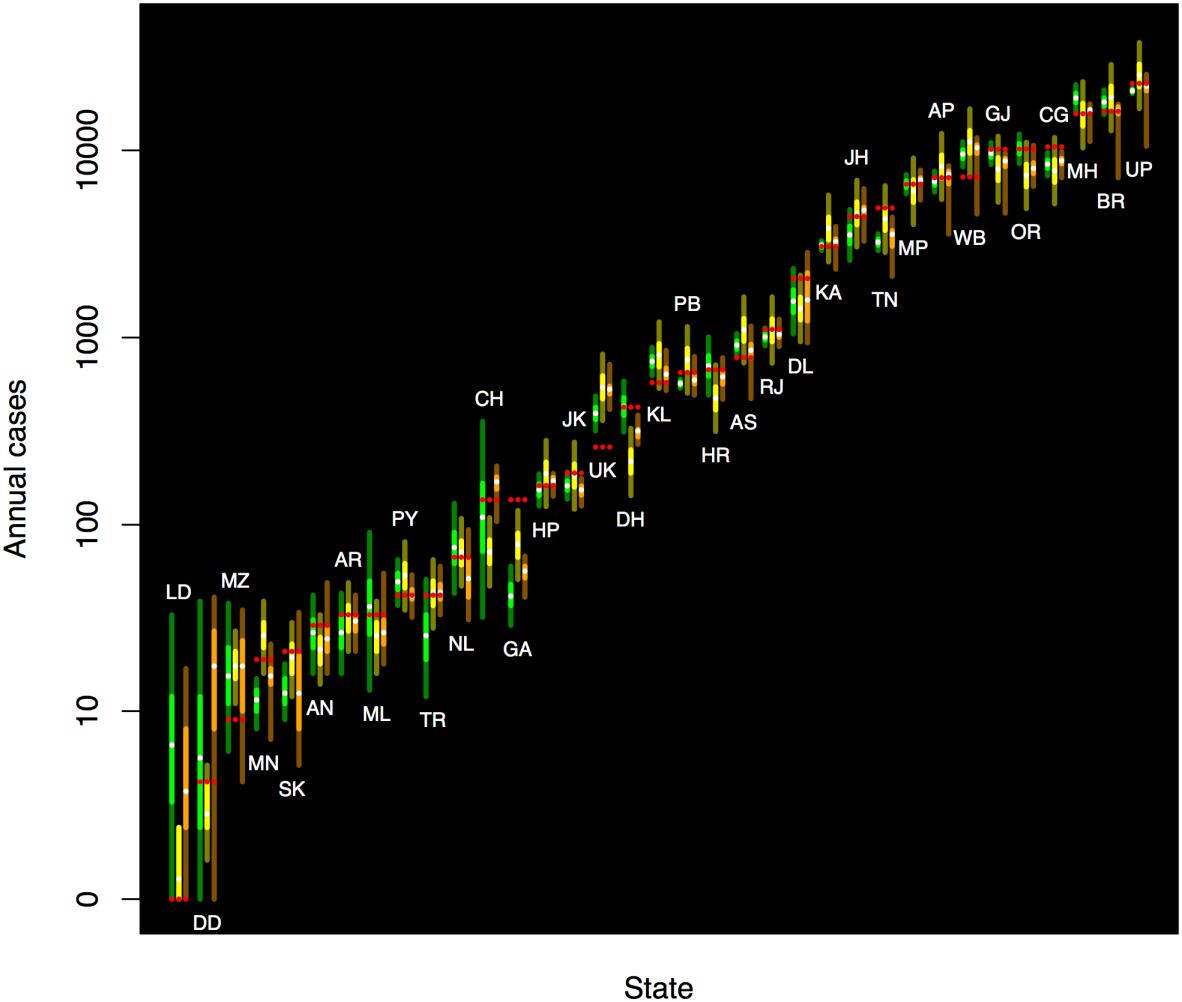
Temporal trends in leprosy for the world, India, Brazil, and Indonesia for 2006–2014, together with forecast distributions for 2015. Temporal trends and regression lines are shown using large dots and dashed lines, for 2014 and before. Forecast distributions are indicated by vertical bands, with green (left) for Holt-Winters, yellow (center) for regression, and orange (right) for expert opinion. The interquartile region is shown in bright green, yellow, and orange, respectively, and above and below, the remainder of the 95 percent central coverage region is indicated in dark green, olive, and brown (respectively). The median forecast for 2015 is shown as a small white dot; the observed data for 2015 is shown as as a small red dot. Distributions were derived from Holt-Winters, regression (ordinary least squares for the world data, linear mixed effects regression for the three countries), and expert survey. The observed counts are shown in red.

Leprosy trends (2006–2014) for the world, and for India, Brazil, and Indonesia are shown in Fig 1. Forecasts were derived from Holt-Winters, regression, and from the expert survey; quantiles of these forecasts are also shown in Fig 1. Table 1 summarizes these forecasts, and Table 2 summarizes the scores. A total of 103 individuals provided forecast responses to at least one of the forecast targets, with a total of 90 individuals providing world forecasts. The number of respondents for Brazil was 82, for India was 87, and for Indonesia was 74. Because each respondent was only shown a maximum of six Indian states or territories, the number of expert responses for each was smaller; the numbers of responses ranged from a minimum of 8 for Andhra Pradesh and Uttar Pradesh, and to a maximum of 16 for Karnataka. (Note that for this forecasting exercise, Andhra Pradesh case counts were combined with the new state of Telengana, for historic consistency.)

**Table 1:** Probabilistic forecasts for leprosy new case detection, world, and India, Brazil, and Indonesia, 2015. We show the mean and standard deviation of probabilistic forecasts using the pooled ensemble of experts, using linear mixed effects regression, and modified Holt-Winters forecasts (*smoothing*), as described in the text.

Location	Expert ensemble	Regression	Smoothing	Observed
World	209940 ± 16570	206719 ± 4925	202215 ± 7342	210758
Brazil	29852 ± 3171	29960 ± 3254	30072 ± 1579	26395
India	124315 ± 10716	121258 ± 13233	120116 ± 6193	127326
Indonesia	16684 ± 1726	17331 ± 1891	17356 ± 1443	17202

**Table 2:** Performance of forecasting methods, determined by mean absolute error and ignorance (log-likelihood) scoring, shown for expert ensemble, regression, and modified Holt-Winters.

Forecast	Expert ensemble		Regression		Holt-Winters	
Target	Abs. Err.	LL	Abs. Err.	LL	Abs. Err.	LL
World	818	-8	4039	-10	8543	-11
Brazil	3457	-10	3565	-10	3677	-11
India	3011	-9	6068	-11	7210	-10
Indonesia	518	-9	129	-8	154	-8

Abs. Err.: absolute error; LL: log-likelihood.

Leprosy trends for the world, and for India, Brazil, and Indonesia for 2006–2014 are shown in Fig 1.

**Fig 2:**
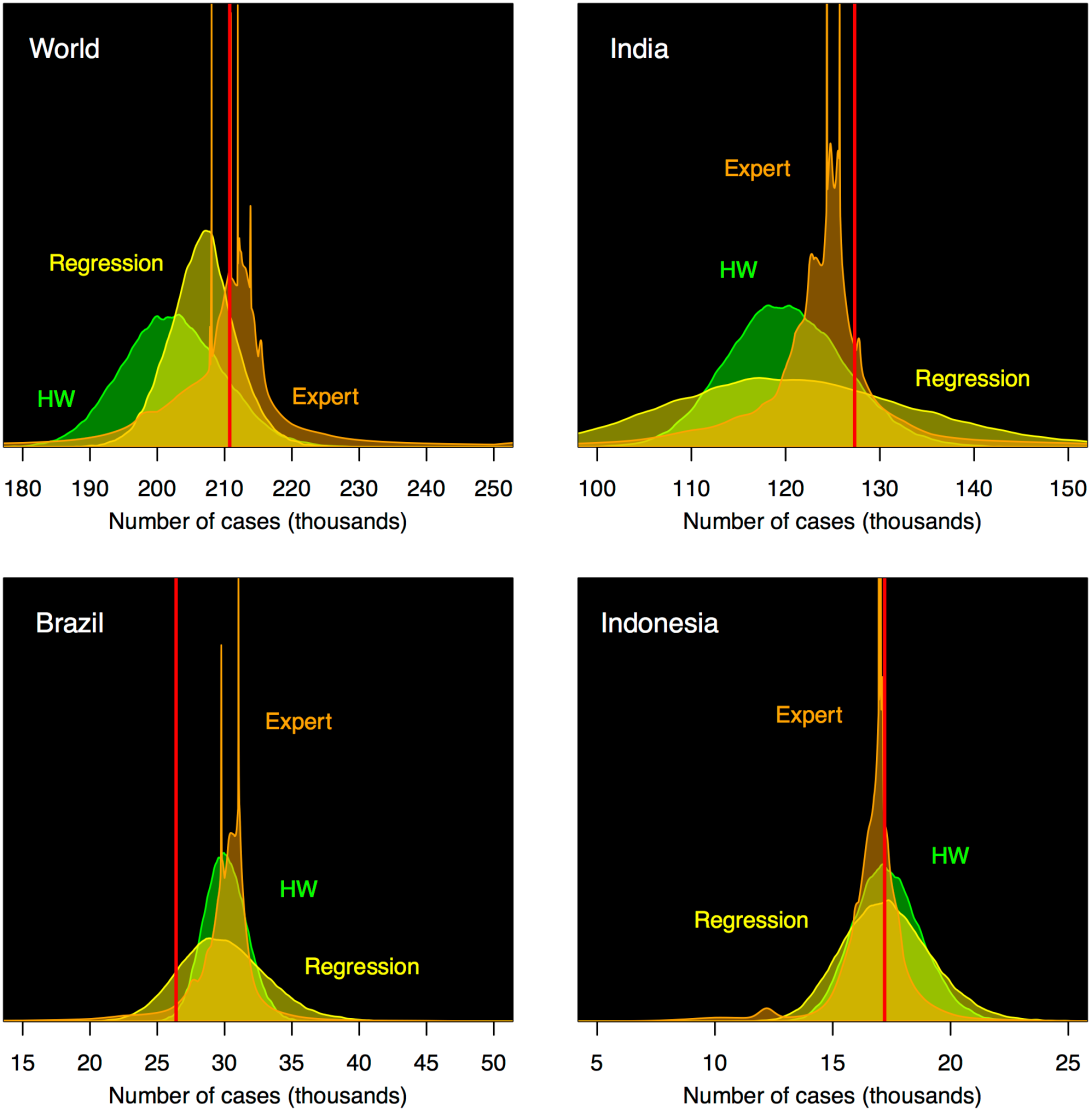
Probabilistic forecasts for the distribution of leprosy cases for the year 2015 for the world, India, Brazil, and Indonesia, derived from experts (orange), regression (yellow), and simple Holt-Winters (green). The vertical axis shows density; the red line indicates where the observed data fell.

Fig 2 shows the pooled (ensemble) forecast (orange), together with the WHO reported count, for the world, India, Brazil, and Indonesia. For comparison, we also show the forecast distribution derived from Holt-Winters (HW, green) and from regression (yellow). The expert ensemble distributions show pronounced spikes, reflecting the small forecast widths given by specific experts. The observed value for the world was 210758, as compared to the mean of the expert ensemble distribution for the world 209940 and the standard deviation 16570.

We compared the individual experts to the ensemble average of all experts. For forecasts of the world reported total, 25.6% of the experts had a lower absolute error than the ensemble mean. Similarly, for Brazil, India, and Indonesia, 28%, 49.4%, and 58.1% achieved a lower absolute error than the respective ensemble mean. For the likelihood scores, we found that a total of 27.8% had a more favorable log-likelihood score than the ensemble forecast. For Brazil and India, 28% and 55.2% achieved a more favorable log-likelihood score than the respective ensemble, while for Indonesia, the ensemble outperformed all the individual experts. Alternative methods to calculate log-likelihood scores from expert elicitations may yield somewhat different findings.

**Fig 3:**
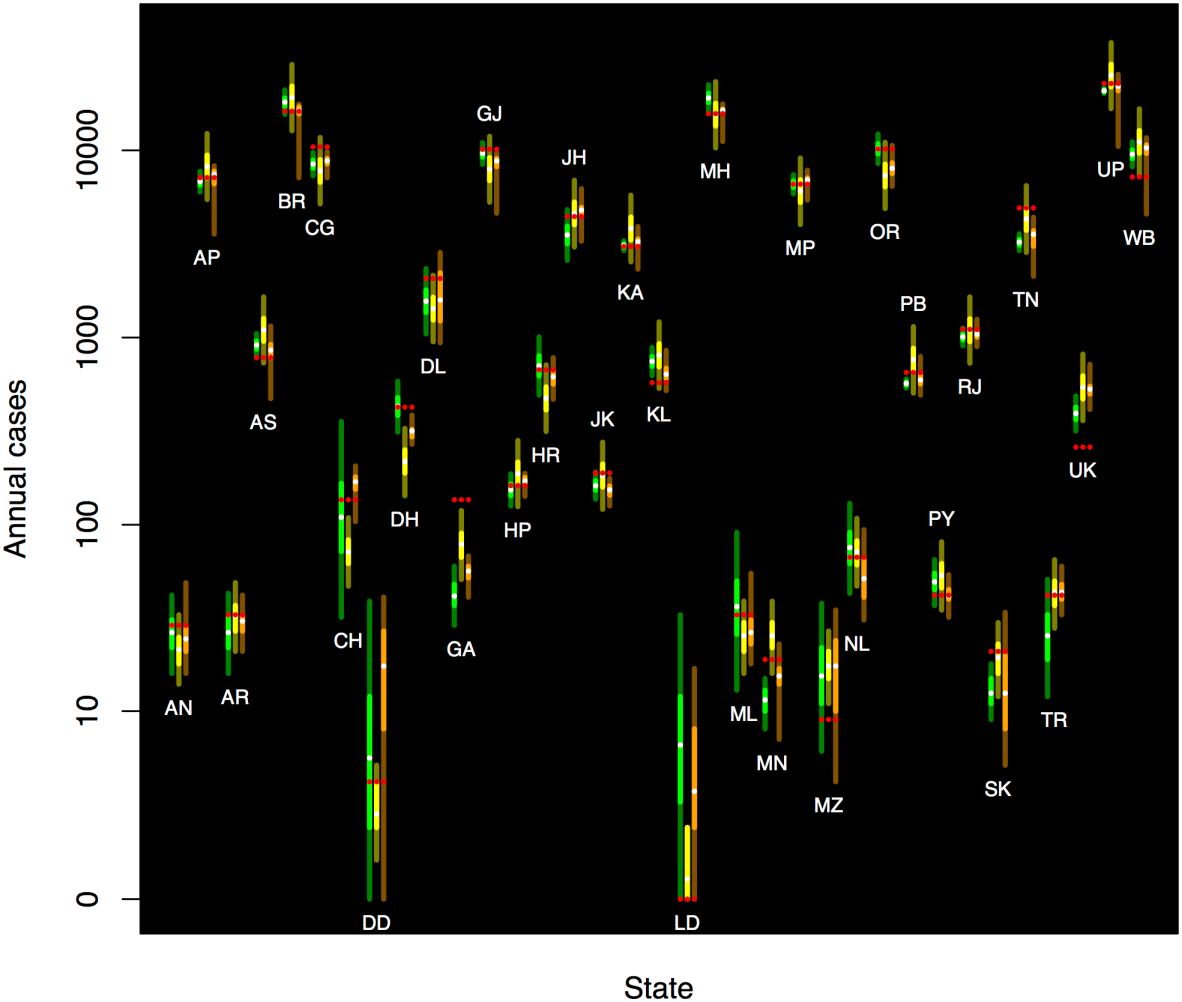
Probabilistic forecasts for the distribution of leprosy cases for the year 2015 for each state and union territory of India derived from experts (orange, left), regression (yellow, central), and simple Holt-Winters (green, right). The median is indicated with a white dot; the bright central band (orange, yellow, green, respectively) corresponds to the interquartile region, and the remainder of the 95 percent central coverage region is indicated by the darker region (brown, olive, dark green, respectively). The observed data for 2015 are shown in red. The pseudologarithm transformation (sinh^−1^(*x*/2)) was used for the vertical axis (asymptotically logarithmic, but finite at zero).

Forecasts for each of the states or territories of India are shown in Table 3, including forecast mean and standard deviation. The forecast distributions are, in general, asymmetric (not shown). Repeated measures ANOVA provides no evidence that any of the three methods yielded a smaller absolute error for the states and territories of India (*P* = 0.79) or a more favorable log-likelihood (ignorance) score (*P* = 0.09). Forecasts for each state using Holt-Winters, regresssion, and the expert ensemble are shown in Fig 3.

**Table 3:** Probabilistic forecasts for leprosy new case detection for the states and territories of India, 2015. We show the mean and standard deviation of probabilistic forecasts using the pooled ensemble of experts, using linear mixed effects regression, and modified Holt-Winters forecasts (*smoothing*), as described in the text.

Location	Expert ensemble	Regression	Smoothing	Observed
Andaman and Nicobar	26 ± 7	23 ± 5	28 ± 6	29
Andhra Pradesh	6938 ± 1244	8374 ± 1758	6823 ± 445	7155
Arunachal Pradesh	31 ± 5	33 ± 7	28 ± 6	33
Assam	848 ± 158	1123 ± 234	914 ± 66	781
Bihar	15468 ± 2936	19546 ± 4091	18146 ± 1391	16185
Chandigarh	167 ± 25	74 ± 16	132 ± 88	136
Chhattisgarh	8687 ± 675	7942 ± 1668	8460 ± 612	10440
Dadra and Nagar Haveli	315 ± 27	223 ± 47	434 ± 69	425
Daman and Diu	19 ± 11	4 ± 1	10 ± 12	4
Delhi	1719 ± 591	1458 ± 306	1597 ± 331	2068
Goa	56 ± 6	80 ± 17	43 ± 8	136
Gujarat	8320 ± 1271	8113 ± 1705	9659 ± 659	10138
Haryana	620 ± 90	485 ± 102	718 ± 131	672
Himachal Pradesh	170 ± 12	192 ± 40	155 ± 15	162
Jammu and Kashmir	153 ± 13	188 ± 40	163 ± 13	189
Jharkhand	4715 ± 652	4703 ± 986	3578 ± 571	4432
Karnataka	3198 ± 365	3910 ± 822	3099 ± 94	3065
Kerala	653 ± 92	822 ± 174	749 ± 66	574
Lakshadweep	6 ± 4	1 ± 0	10 ± 9	0
Madhya Pradesh	6836 ± 609	6193 ± 1296	6608 ± 394	6597
Maharashtra	15845 ± 1604	15841 ± 3309	19123 ± 1595	15695
Manipur	16 ± 3	26 ± 6	12 ± 2	19
Meghalaya	30 ± 9	26 ± 6	41 ± 20	33
Mizoram	19 ± 9	18 ± 4	18 ± 8	9
Nagaland	55 ± 16	73 ± 15	79 ± 22	67
Odisha	8129 ± 1016	7499 ± 1566	10236 ± 945	10174
Puducherry	43 ± 5	55 ± 12	50 ± 7	42
Punjab	603 ± 88	778 ± 162	569 ± 16	651
Rajasthan	1051 ± 120	1118 ± 235	1010 ± 56	1106
Sikkim	16 ± 8	20 ± 4	13 ± 2	21
Tamil Nadu	3424 ± 525	4396 ± 924	3235 ± 170	4925
Tripura	45 ± 6	45 ± 9	27 ± 10	42
Uttar Pradesh	20647 ± 4118	25647 ± 5358	20811 ± 366	22777
Uttaranchal	536 ± 65	554 ± 117	396 ± 43	260
West Bengal	9709 ± 1891	11335 ± 2377	9556 ± 750	7211

We examined several predictors of the absolute error score. No substantial differences were found in the absolute error score using the elicited probability of achieving global “interruption of transmission” by 2020, the elicited probability of reducing the incidence of new leprosy-related grade 2 disability to less than 1 per million by 2020, to choosing India or Brazil as the country for which the for which the respondent has greatest expertise, having a medical degree, or correctly answering the expertise assessment question. A higher elicited probability of achieving success was statistically associated with a slightly higher error score (i.e. “optimists” did slightly worse), though the estimated magnitude of this effect was small. Selected estimates are provided in Table 4. The first two rows of the table show the effect of changes in elicited probabilities for global interruption or achieving the disability targets; roughly, the more optimistic the respondent is (higher elicited success probability), the higher the error scores for the world, for India, and for Indonesia, but the lower the error scores for Brazil. The second two rows of the table exhibit no convincing evidence that self-reported country-specific expertise is a statistically significant predictor of absolute error score (after considering correction for multiple comparisons). We also found no evidence that a medical degree or a correct answer on the assessment question had any relation to absolute error score.

**Table 4:** Analysis of absolute error score for world, Brazil, India, and Indonesia. Columns refer to overall forecast error in predicted number of cases for the world, Brazil, India, and Indonesia. Row variables refer to selected univariate predictors, as described in the text. Summary effect sizes reported here are pseudomedians for binary predictors, or Spearman rank correlations for continuous predictors.

	World	Brazil	India	Indonesia
Global Interruption^†^	0.03	-0.29	0.34	0.34
	(-0.17, 0.2)	(-0.48, -0.1)	(0.11, 0.5)	(0.11, 0.5)
	*P* = 0.92	*P* = 0.32	*P* = 0.016	*P* = 0.01
Disability Reduction^†^	0.05	-0.21	0.15	0.27
	(-0.17, 0.2)	(-0.42, 0)	(-0.05, 0.4)	(0.07, 0.5)
	*P* = 0.92	*P* = 0.3	*P* = 0.21	*P* = 0.019
India Expertise*	-263.81	500	-1364.61	-471.71
	(-1828.75, 1192.2)	(0, 1091.9)	(-4039.85, 0)	(-900, -25)
	*P* = 0.68	*P* = 0.1	*P* = 0.034	*P* = 0.012
Brazil Expertise*	-355.57	-183.66	0	60.61
	(-2234.2, 1500)	(-1000, 374.4)	(-1409.21, 1530.7)	(-183.66, 281.7)
	*P* = 0.66	*P* = 0.48	*P* = 0.9	*P* = 0.37

* Summary effect measure: pseudomedian.

^†^ Summary effect measure: Spearman rank correlation.

## Discussion

Experts in leprosy control were asked to provide short term forecasts of leprosy for the world, for the top three reporting countries, and for states of India. These forecasts were scored probabilistically and compared with statistical approaches. These forecasts were simply for the next reporting period, an application which we expected to be relatively undemanding. Forecasts further into the future, or which are conditional on policy changes, were not considered.

All methods performed essentially equivalently. Individual experts exhibited considerable variability, and showed narrow forecast intervals. Individually, the experts occasionally performed poorly, but the entire ensemble of experts showed similar skill to the statistical approaches. We note that logistical limitations in the survey rendered it impossible to elicit a large number of forecasts suitable for formal statistical comparison of the experts with the statistical models regarding forecast skill. Moreover, for very short term forecasts, even simple statistical procedures may be expected to produce adequate performance. Our results suggest that short term forecasts for leprosy, a slow disease with a long incubation period, may be adequately rendered by an ensemble of experts or using relatively simple statistical approaches. Expert opinion could conceivably far outperform statistical methods under circumstances in which epidemiological or surveillance knowledge would be valuable. For example: (1) human experts might be less likely than a statistical model to extrapolate a large rising trend in leprosy annual new case detection rates over several years (on the basis that improved case detection may be a better explanation than an actual leprosy epidemic), (2) human experts might be less likely than a statistical model to be misled by a sudden change in case counts, (3) human experts could use knowledge regarding changes in active case finding or reporting known to be taking place on the ground even before these have produced any changes in surveillance data (such as the enhanced case detection policy in selected districts in India in 2012), and (4) human experts could use other sources of data, such as weather or political changes, that may be important. Expert opinion forecasting over longer time periods or for more and smaller geographic regions could provide greater statistical power in the future to detect such effects, if present. We also note that experts, as individuals, did not perform well, in large part due to an excessively small forecast variance. In public health, further work is needed to understand the limitations of expert forecasting for longer time horizons or for counterfactual settings. Unfortunately, expert opinion can also suffer from well-known cognitive biases [38] or from real or perceived economic or political interests. Statistical or mathematical methods for forecasting leprosy [39] may, within limits, provide greater openness and transparency, helping us understand what data and what structural features determine the value of a forecast. We anticipate that an ensemble-based forecasting algorithm that combines each method may yield better performance than each individual forecasting method (e.g. [40, 41]).

Far fewer cases are reported today than in previous decades [36], and stated world prevalence targets were achieved globally in 2000 [42]. Despite these past successes, recent years have seen some slowing or stalling in leprosy control, and some express considerable skepticism regarding data accuracy [12, 43–45]. It is claimed that leprosy was “eliminated as a public health problem” globally in 2000 with the formal achievement of stated world prevalence targets [42]. Leprosy may be a far smaller public health problem than in the past, but resources and infrastructure are still required for its control and for the prevention of needless disease and disability among those still infected.

Can probabilistic forecasting help? In the overall setting of public health, the ability to predict future trends, even if only qualitatively, is necessary to make sound policy recommendations. If past skill in forecasting can be shown, and if valid data are used, then evaluation of probabilistic forecasts can provide support for such recommendations. Such efforts are important in helping earn, and not merely request, the public’s trust. Trust, when lost in public health, is not easily regained, and such loss can have unfortunate consequences (e.g. [46–50]). We believe probabilistic forecasting offers public health an opportunity to take a leading role in such institutional assessments.

## Appendix

We used the following searches in PubMed:

- forecast + (influenza or HIV or malaria or tuberculosis or measles or ebola)
- predict + incidence + (influenza or HIV or malaria or tuberculosis or measles or ebola) and does not contain predictor
- predict + prevalence + (influenza or HIV or malaria or tuberculosis or measles or ebola) and does not contain predictor

This provides us with a selection of individuals with published expertise in forecasting infectious diseases, but does not attempt to be comprehensive.
