# Correction: Short-term leprosy forecasting from an expert opinion survey

**DOI:** 10.1371/journal.pone.0189976

**Published:** 2017-12-14

**Authors:** Michael S. Deiner, Lee Worden, Alex Rittel, Sarah F. Ackley, Fengchen Liu, Laura Blum, James C. Scott, Thomas M. Lietman, Travis C. Porco

Fig 1 is incorrect. The image that appears as Fig 1 should be Fig 3. Please see the correct Figs 1 and 3 here.

**Fig 1:**
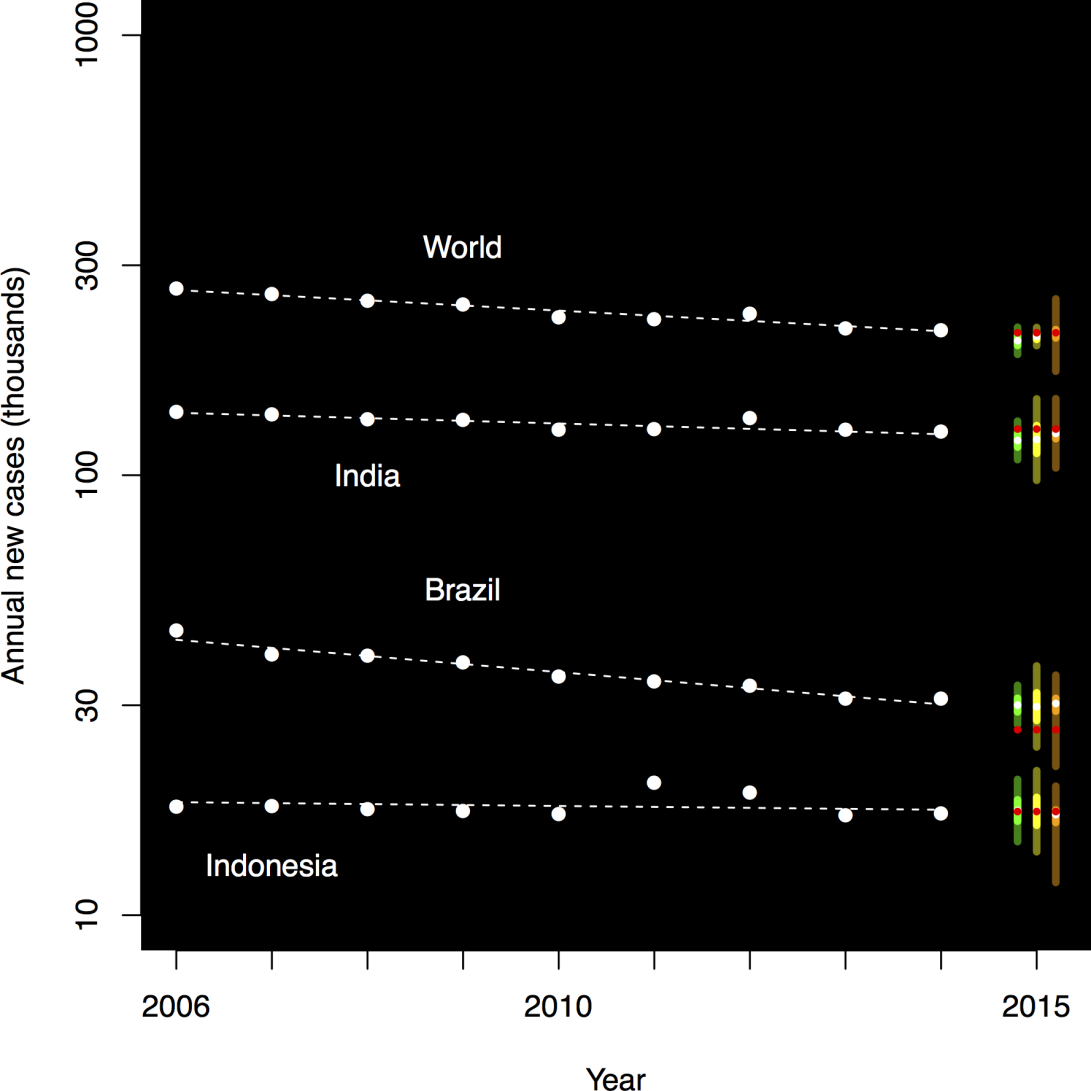
Temporal trends in leprosy for the world, India, Brazil, and Indonesia for 2006–2014, together with forecast distributions for 2015. Temporal trends and regression lines are shown using large dots and dashed lines, for 2014 and before. Forecast distributions are indicated by vertical bands, with green (left) for Holt-Winters, yellow (center) for regression, and orange (right) for expert opinion. The interquartile region is shown in bright green, yellow, and orange, respectively, and above and below, the remainder of the 95 percent central coverage region is indicated in dark green, olive, and brown (respectively). The median forecast for 2015 is shown as a small white dot; the observed data for 2015 is shown as a small red dot. Distributions were derived from Holt-Winters, regression (ordinary least squares for the world data, linear mixed effects regression for the three countries), and expert survey. The observed counts are shown in red.

**Fig 3:**
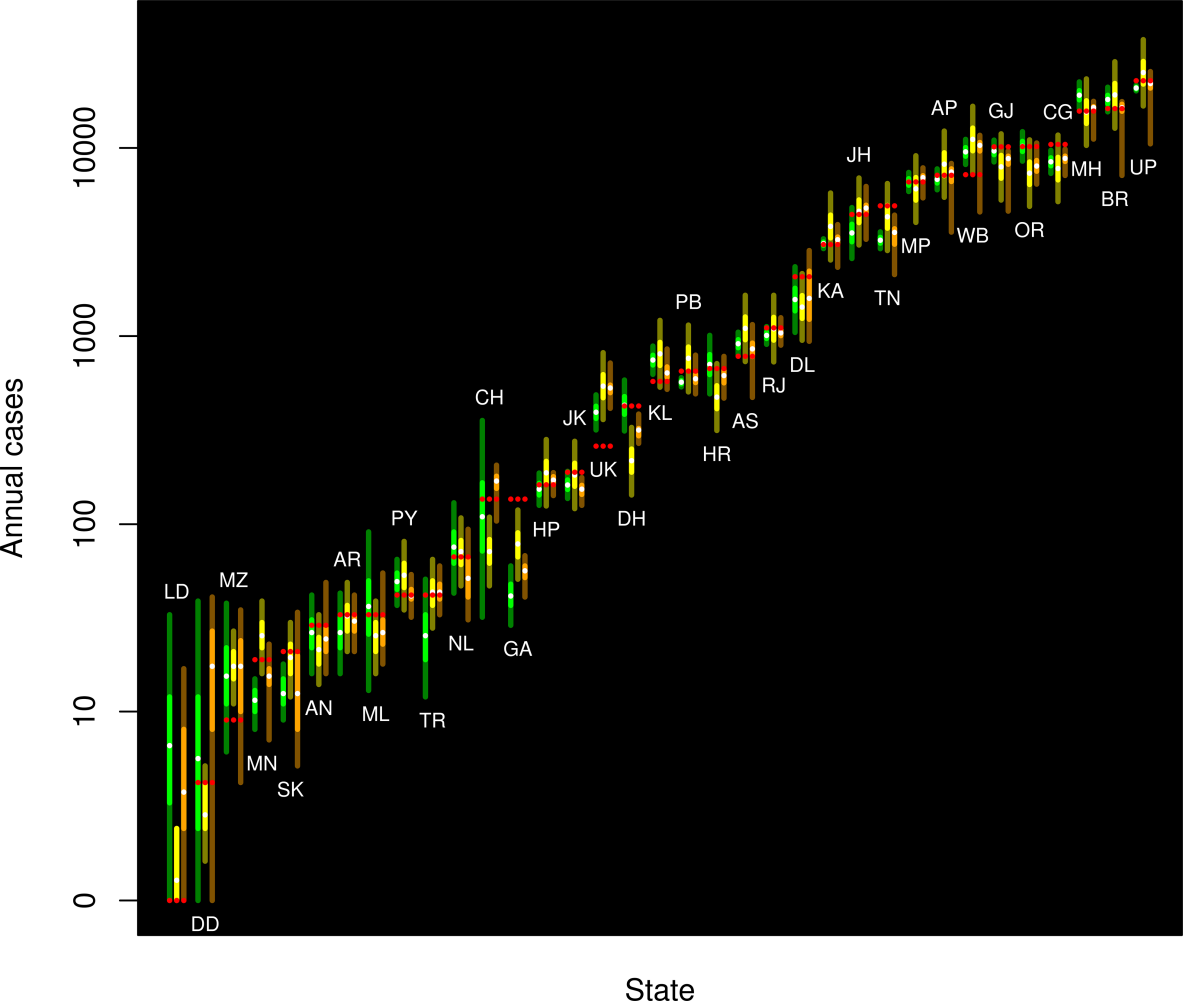
Probabilistic forecasts for the distribution of leprosy cases for the year 2015 for each state and union territory of India derived from experts (orange, left), regression (yellow, central), and simple Holt-Winters (green, right). The median is indicated with a white dot; the bright central band (orange, yellow, green, respectively) corresponds to the interquartile region, and the remainder of the 95 percent central coverage region is indicated by the darker region (brown, olive, dark green, respectively). The observed data for 2015 are shown in red. The pseudologarithm transformation (sinh^−1^(x/2)) was used for the vertical axis (asymptotically logarithmic, but finite at zero).
